# Supplementary material for: Impact of Metastatic Pattern on Survival in Patients with Posterior Uveal Melanoma: A Retrospective Cohort Study
Source: Cancers (Basel). 2024 Sep 30;16(19):3346. doi: 10.3390/cancers16193346 (PMC11475269; doi:10.3390/cancers16193346)
Supplement: Supplementary file 1 [file cancers-16-03346-s001.zip › TableS1.pdf]

Patient characteristics of the excluded patients

	Total	(%)
**Patients, n**	71	
**Age at metastatic diagnosis (year)**		
Median	69.0 (IQR 61.0-76.0)	
**Sex, n**		
Women	32	45
Men	39	55
**Hepatic involvement, n**		
Known hepatic metastases	60	85
Hepatic involvement unknown	11	15
**Treatment**		
No ipi+nivo	70	99
Ipi+nivo	1	1
**Median overall survival (months)**	2.3 (IQR 0.8 – 8.6 months)	
**Median DFI (months)**	24.5 (IQR 15.0-48.0)	

Table S1. Patient characteristics of the 71 excluded patients with metastatic posterior uveal melanoma.

Abbreviations: DFI, disease-free interval; ipi+nivo, first-line treatment with the combination of ipilimumab and nivolumab; IQR, interquartile range.
