# Supplementary figures and images for: Impact of Metastatic Pattern on Survival in Patients with Posterior Uveal Melanoma: A Retrospective Cohort Study
Source: Cancers (Basel). 2024 Sep 30;16(19):3346. doi: 10.3390/cancers16193346 (PMC11475269; doi:10.3390/cancers16193346)

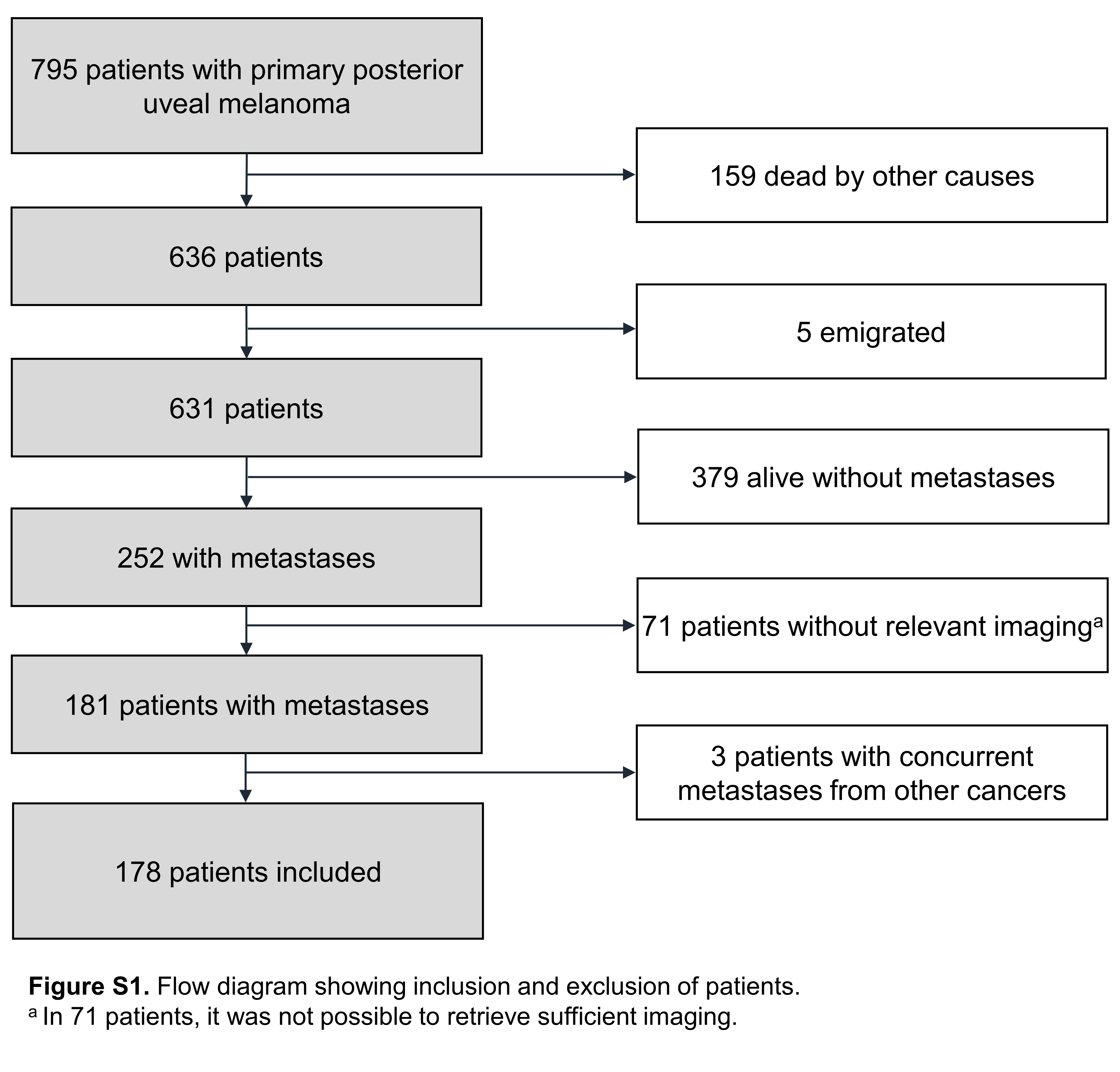

Supplement: Supplementary file 1 [file cancers-16-03346-s001.zip › Figs1.tif]
